# Passive Frequency Tunability in Moiré-Inspired Frequency Selective Surfaces Based on Full-Wave Simulation

**DOI:** 10.3390/mi16060702

**Published:** 2025-06-12

**Authors:** Jieun Hwang, Sungcheol Hong

**Affiliations:** 1 Department of Physics, Yonsei University, Seoul 03722, Republic of Korea; 2024324016@yonsei.ac.kr; 2 Department of Electronic & Electrical Convergence Engineering, Hongik University, Sejong 30016, Republic of Korea

**Keywords:** frequency-selective surfaces, Moiré pattern, passive tunability, full-wave simulation, metamaterials, bandstop filter, electromagnetic surface design

## Abstract

This paper presents a simulation-based investigation of passive frequency tunability in frequency-selective surfaces (FSSs) enabled by Moiré pattern interference. By overlapping two identical hexagonal FSS layers and introducing rotational misalignment between them, we demonstrate that the resulting Moiré patterns induce significant shifts in the resonance frequency without any external bias or active components. Using full-wave simulations in HFSS, we show that rotating the second layer from 0° to 30° can shift the resonant frequency from 4.4 GHz down to 1.2 GHz. This tunable behavior emerges solely from geometrical manipulation, offering a low-complexity alternative to active tuning methods that rely on varactors or micro-electromechanical systems (MEMSs). We discuss the theoretical basis for this tuning mechanism based on effective periodicity modulation via rotational interference and highlight potential applications in passive reconfigurable filters and refractive index sensors. The proposed approach provides a promising route for implementing tunable electromagnetic structures without compromising simplicity, power efficiency, or integration compatibility.

## 1. Introduction

Frequency-selective surfaces (FSSs) are periodic structures that exhibit selective transmission or reflection of electromagnetic waves at specific frequency bands [1,2]. Due to their inherent filtering characteristics, FSSs have found broad applications in antennas, electromagnetic shielding, wireless communication systems, and sensing technologies. Traditional FSS designs rely on fixed geometric patterns that determine their resonance frequencies, often making them static and inflexible once fabricated [3].

To address the limitation of fixed resonance, numerous approaches have been developed for tunable FSSs. These include integration with varactor diodes, PIN switches, micro-electromechanical system (MEMS) actuators, and phase-change materials [4,5]. While such methods allow dynamic frequency control, they introduce complexity in terms of external biasing, circuit integration, and increased power consumption. Additionally, many active tuning approaches scale poorly when extended to large-area FSS arrays, making them less suitable for applications requiring simplicity, low power, or passive operation [6,7].

**Figure 1:**
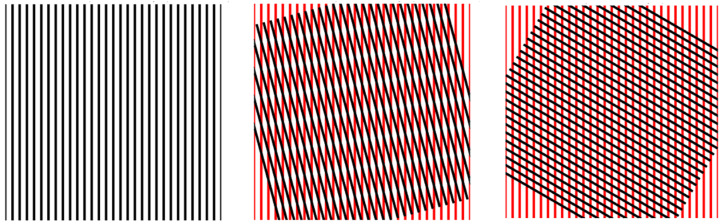
Example of a Moiré pattern, when two sets of stripes are overlaid, changing the angle of one layer causes the pattern within to distort or shift.

Recent research has explored geometric and structural modifications as alternatives to active control. Among these, multilayered or misaligned periodic structures have demonstrated promise in enabling electromagnetic tunability without relying on external circuitry. In particular, Moiré patterns—interference effects arising from the superposition of two periodic grids with slight rotational or scale differences (Figure 1)—have attracted attention due to their ability to generate new effective periodicities. Moiré phenomena have been extensively studied in optical imaging, mechanical sensors, and two-dimensional materials such as graphene, where twist-induced superlattices exhibit novel electrical and optical properties [8,9].

Motivated by these observations, this work investigates a passive frequency tuning method based on Moiré interference in multilayered FSSs. By overlapping two identical hexagonal FSS layers and rotating one of them with respect to the other, a dynamic geometric modulation is introduced, resulting in a shift in the effective resonance frequency. This approach requires no active components, no biasing circuitry, and no mechanical deformation, offering a low-complexity path toward tunable electromagnetic surfaces.

**Figure 2:**
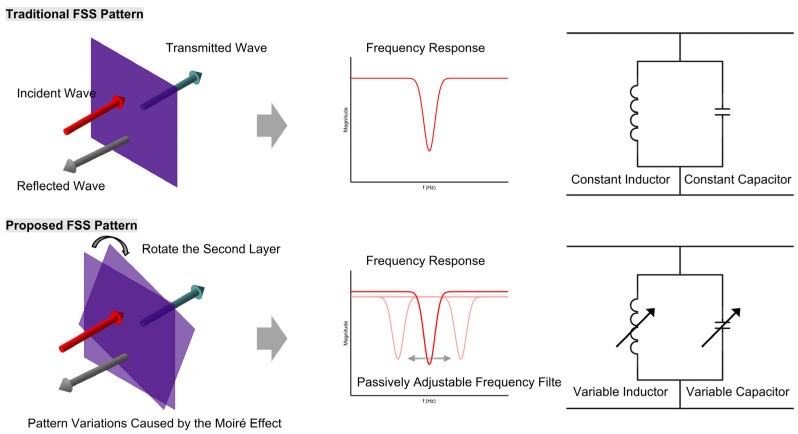
A comparison between a traditional FSS with fixed frequency characteristics and a Moiré pattern-based FSS with tunable frequency response and their corresponding equivalent circuits.

In this study, we present full-wave simulation results using a commercial electromagnetic solver to analyze the frequency response of Moiré-inspired FSSs under different rotational misalignments. Our goal is to validate the hypothesis that Moiré-induced geometric reconfiguration can serve as a viable mechanism for passive frequency tuning (Figure 2). We further discuss the theoretical basis for such behavior and explore its relevance to future applications in optical filtering, biosensing, and reconfigurable metasurfaces [10,11,12].

## 2. Theory and Background

Moiré patterns arise when two periodic structures are overlaid with slight rotational or translational offsets [13]. This geometric interference results in a new, larger-scale pattern whose periodicity differs from that of the individual layers. The phenomenon is widely observed in optics, materials science, and imaging, often producing visually striking effects due to spatial aliasing [14].

### 2.1. Moiré Interference and Effective Periodicity

When two identical periodic grids with period P are superimposed with a relative rotation angle θ, the resulting Moiré pattern forms a new periodic structure with an effective period $P_{eff}$ that is typically much larger than P [15]. This effective periodicity can be approximated in certain cases using [15,16]:

(1) Peff≈ P2sin⁡θ2

As θ increases, the Moiré fringes become denser, reducing the effective periodicity. Conversely, for small angles, the effective periodicity can become significantly larger, leading to long-range interference patterns. This modulation in spatial frequency forms the core mechanism for the proposed tunability in our frequency-selective surface design.

### 2.2. Frequency-Selective Surfaces and Resonance Behavior

FSS structures behave similarly to filters, exhibiting transmission or reflection at specific frequencies determined primarily by the geometry of their unit cells and their periodic arrangement [3]. In the case of a simple planar array with lattice constant a, the resonance condition for a typical FSS is influenced by

- The effective inductance and capacitance of the pattern [17];
- The lattice periodicity, which affects phase accumulation [18];
- The substrate properties, such as dielectric constant [19].

In many planar FSS designs, the resonance frequency $f_{0}$ can be roughly estimated from the geometric and material parameters using [18,20]:

(2) $$f_{0}\propto \;\frac{1}{\sqrt{LC}}\;\propto \;\frac{c}{a_{eff}}\;$$

where $a_{eff}$ is the effective periodicity, and c is the speed of light in the medium. A decrease in effective periodicity $a_{eff}$ leads to an increase in the resonant frequency, and vice versa. Thus, by introducing a Moiré pattern and adjusting the rotation angle θ, one can dynamically control the effective periodicity of the superimposed FSS layers, thereby tuning the resonance behavior without modifying the physical size or shape of individual unit cells [1,2,16].

From an equivalent circuit perspective, the rotational misalignment increases both the effective inductance, due to longer current paths, and the effective capacitance, due to changes in overlapping coupling areas between adjacent conductors. This leads to a monotonic decrease in the resonance frequency, following this relation:

(3) $$f_{0}\propto \;\frac{1}{\sqrt{L_{\theta }\cdot C_{\theta }}}$$

As the rotation angle θ increases, the effective current path length becomes longer, resulting in increased inductance. This behavior can be approximated by a linear model:

(4) $$L_{\theta }\approx \;L_{0}\cdot (1+\alpha \cdot \theta )\;$$

Simultaneously, the overlapping area between adjacent conductors changes as the layers rotate, affecting the coupling capacitance. Although this change is generally nonlinear, it can be approximated in a first-order model as

(5) $$C_{\theta }\approx \;C_{0}\cdot (1+\beta \cdot \theta )\;$$

Here, $L_{0}$ and $C_{0}$ represent the inductance and capacitance at the rotation angle of 0°, while $L_{\theta }$ and $C_{\theta }\;$ denote the inductance and capacitance at a given rotation angle θ. The parameters α and β are geometry-dependent constants. Thus, as the rotation angle θ increases, both the inductance (due to the extended current path) and the capacitance (due to varying coupling area) increase, which together result in a lower resonance frequency.

### 2.3. Advantages of Moiré-Tuned Passive Structures

Unlike actively tunable FSS architectures that rely on bias-controlled varactors, diodes, or MEMS components, Moiré-tuned designs achieve frequency reconfigurability through purely geometric means. This provides several key advantages:

- No external power supply or control lines required [16];
- Mechanically simple implementation via rotation or shifting [21];
- Compatible with large-area, passive systems [22];
- Potential for miniaturization and integration with optical or RF platforms [23].

These theoretical insights form the basis for the passive tuning approach investigated in this study. The following sections present a simulation-based analysis of Moiré-patterned FSSs and their resulting frequency response under controlled angular rotation.

## 3. Materials and Methods

### 3.1. Unit Cell Design

**Figure 3:**
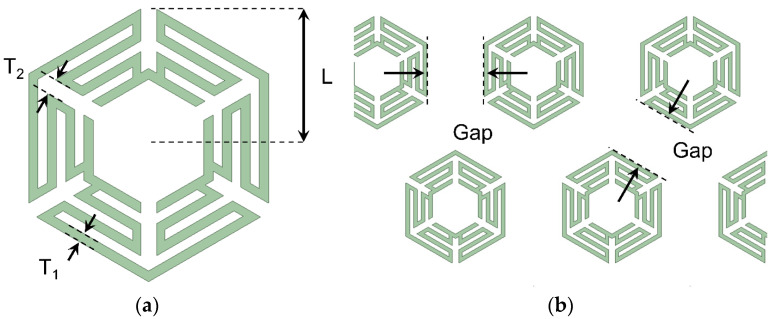
(**a**) FSS unit cell pattern and dimensions. (**b**) Distance between cells.

The unit cell employed in this study is based on a hexagonal loop geometry incorporating meander lines to enhance inductive loading. This design was chosen to reduce the resonant frequency while maintaining a compact footprint suitable for integration into multilayer structures. The rotational symmetry of the hexagonal shape ensures polarization-insensitive electromagnetic response under normal incidence, regardless of the linear polarization orientation [24,25,26]. The key geometric parameters are as follows: The side length of each hexagonal unit is 20 mm, the meander line width is approximately 1.33 mm, and the spacing between adjacent meander traces is set to 2.67 mm. The overall cell spacing, or pitch, is also 20 mm, which defines the lattice periodicity across the two-dimensional array (Figure 3).

Two identical layers of this hexagonal FSS are stacked to form the proposed Moiré configuration. The upper layer is rotated relative to the lower one to generate Moiré interference patterns. In this study, rotation angles of 0 degrees, 15 degrees, and 30 degrees are considered to observe their influence on the frequency response. The two layers are separated by a 1 mm air gap, and no additional dielectric layers, control circuits, or electronic components are introduced. The concept aims to demonstrate frequency tunability through geometric alignment alone, thereby offering a fully passive and mechanically simple configuration [21].

### 3.2. Simulation Environment

**Figure 4:**
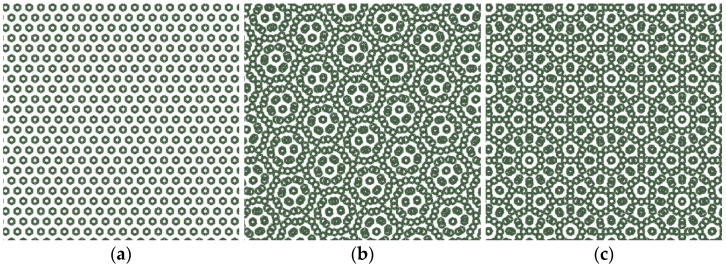
Moiré pattern that is observed when rotating the second layer: (**a**) 0-degree rotation, (**b**) 15-degree rotation, (**c**) 30-degree rotation.

Full-wave electromagnetic simulations were performed using Ansys HFSS (Ansys Electromagnetics Suite 2020 R2-HFSS, Ansys, Canonsburg, PA, USA) to evaluate the frequency response of the proposed FSS configurations [1,2]. The simulation model used the Driven Modal solver, and the boundary conditions were set as radiation boundaries on all sides to emulate an open-space environment. Because the Moiré structure introduces non-periodicity through layer rotation, it was not feasible to apply Floquet ports or master/slave boundary conditions, which are typically used in periodic array simulations. Instead, a finite 25-by-25 array of unit cells was modeled to approximate realistic field interactions while keeping computational requirements within a manageable range (Figure 4).

The conductive traces were modeled as perfect electric conductors (PECs) to represent ideal metallic behavior, and the separation between the two layers was treated as an air gap. The frequency sweep was set from 0.5 to 6 GHz to fully capture any potential resonance shifts caused by the rotational offset. To ensure accurate simulation results, mesh refinement was applied throughout the structure, especially near the edges and intersections of the meandered traces. From the simulation, the S-parameters (specifically S11) were extracted to analyze the resonance behavior. The primary focus was to determine whether rotation-induced Moiré effects could serve as an effective mechanism for passive frequency tuning, and if so, to quantify the magnitude and direction of the tuning range.

## 4. Results

**Figure 5:**
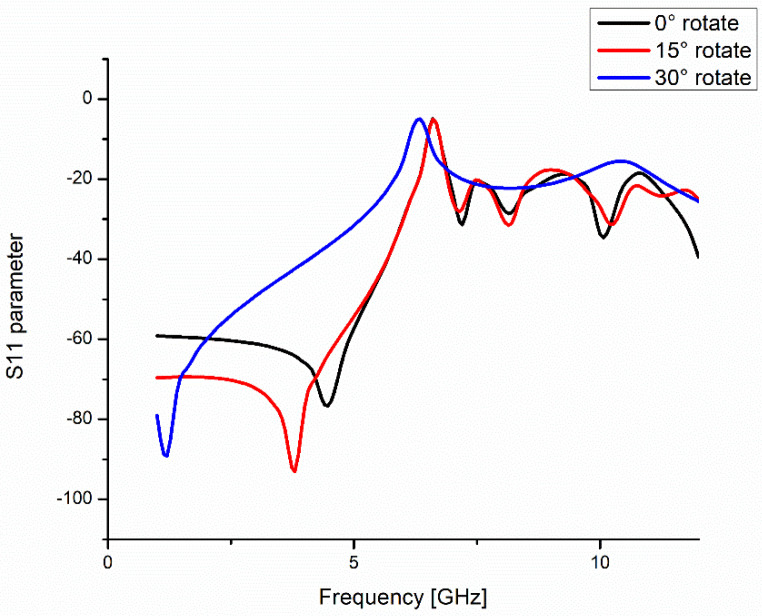
S11 parameter results from HFSS corresponding to Moiré patterns at different angles.

To verify the impact of Moiré patterning on the electromagnetic response of the frequency-selective surface, simulations were conducted for three different rotation angles of the upper FSS layer: 0°, 15°, and 30°. The resulting S-parameter (S11) plots revealed distinct shifts in the resonance frequency as the relative orientation between layers changed. At 0°, where the two layers are perfectly aligned, the structure exhibited a resonance at approximately 4.4 GHz. As the rotation angle increased to 15°, the resonance shifted to 3.8 GHz. At 30°, the structure displayed a pronounced resonance at approximately 1.2 GHz (Figure 5). These results confirm that the effective periodicity of the superimposed layers—governed by the Moiré interference—modulates the electromagnetic resonance characteristics without any alteration to the individual unit cell geometry [20,21]. 

This behavior can be attributed to the rotational modulation of the spatial distribution of current paths and capacitive coupling across the two layers. As the overlap between conductive paths changes with increasing rotation angle, the structure supports longer or more distributed current loops, which in turn lower the resonance frequency [27]. From an effective circuit model perspective, this corresponds to an increase in the equivalent inductance and capacitance of the composite surface [28]. The relationship between geometric rotation and frequency shift is nonlinear but monotonic, providing a means to continuously tune the filtering properties of the surface through purely mechanical alignment [29].

The sharpness of each resonance peak was also assessed qualitatively to estimate the effective Q-factor of the structure. At 0°, the resonance peak was relatively broad, indicating a lower Q-factor, whereas at 30°, the resonance was more confined in frequency, suggesting an increase in frequency selectivity. Although this study did not include a formal calculation of the quality factor, the narrowing of the S11 bandwidth in higher rotation angles points to improved spectral resolution, which is desirable in filtering and sensing applications [30].

Overall, these results validate the central hypothesis of this work: that Moiré-induced geometric reconfiguration offers a viable and passive mechanism for electromagnetic frequency tuning [21]. The simulation findings support the potential use of this approach in scenarios where tunability, low complexity, and low power consumption are simultaneously required. In particular, the ability to shift resonance frequencies over a wide range using only mechanical rotation introduces new possibilities for applications in reconfigurable filters, spectral sensors, and integrated photonic or RF platforms [31]. These results also suggest that further optimization of the unit cell geometry and dielectric spacing could enhance the tuning resolution and bandwidth control, providing a pathway toward practical implementation in future systems [7].

## 5. Discussion

The passive frequency tuning behavior demonstrated in this study opens promising opportunities for applying Moiré-inspired FSS designs in practical electromagnetic systems [32]. The ability to dynamically adjust the resonant frequency without requiring any active components, external power sources, or material reconfiguration makes this approach particularly attractive in applications where simplicity, stability, and energy efficiency are paramount [33,34].

One of the most direct applications of the proposed structure lies in optical or microwave filtering. In conventional tunable filters, dynamic control often relies on the integration of varactors, MEMSs, or phase-change materials, each of which introduces additional design complexity, potential reliability concerns, and increased power consumption [35,36,37]. In contrast, the Moiré-tuned FSS structure achieves comparable tuning functionality using only passive geometric modulation. This allows for the development of reconfigurable filters with minimal circuit overhead, especially suitable for embedded or miniaturized systems [34].

Additionally, the structure holds potential for biosensing applications. In refractive index-based sensing, small shifts in resonance frequency can indicate changes in the surrounding medium, such as the presence of specific biomolecules or variations in tissue properties [38,39,40,41,42]. The demonstrated frequency sensitivity to geometric configuration implies that similar shifts could be observed in response to environmental changes, particularly if the Moiré structure is integrated with a sensing interface layer. While the current work does not explicitly simulate such variations, the underlying principle of resonance modulation by spatial distribution is consistent with many surface-based sensing mechanisms [43,44].

The approach may also be extended to spatial modulation in beamforming systems, spectral imaging, or wavefront shaping in metasurface-based optical systems [45,46]. By controlling the relative rotation between layers, one could implement tunable angular selectivity or polarization-sensitive structures. Furthermore, integration with micro-rotating platforms or smart materials could potentially enable dynamic reconfiguration in situ, further expanding the range of practical applications [47,48,49,50]. Moreover, while this study specifically investigates hexagonal loop structures within the microwave band, the general principle of Moiré-based geometric tunability has the potential to be extended to other FSS configurations and frequency regimes, such as the terahertz range, and can also be adapted for optical applications. The approach may also be applicable to various alternative patterns and combinations, such as cross-shaped structures and square ring configurations. In the present study, the interlayer gap was fixed at 1 mm; however, this parameter can also be treated as a tunable variable in future studies. Furthermore, exploring multilayer configurations may open additional research directions for more advanced tunable electromagnetic structures.

Despite the potential, several limitations must be acknowledged. First, the present study is limited to full-wave simulations and does not include experimental validation. The lack of Floquet ports due to the non-periodic nature of the Moiré geometry forced the use of finite array models, which may differ from infinitely periodic behavior [51,52]. Additionally, mechanical rotation at small scales may pose fabrication and alignment challenges, especially in optical or nanophotonic implementations [32,53,54]. Future work could address these issues by exploring scalable fabrication techniques, optimizing layer spacing and dielectric properties, and integrating tunable Moiré structures into real-world devices.

Nevertheless, as a simulation-based proof of concept, this study provides compelling evidence that Moiré patterning offers a simple yet powerful tool for passive electromagnetic tuning. By combining geometric control with high flexibility and integration compatibility, this method contributes to the broader effort of developing efficient, low-cost, and reconfigurable electromagnetic components.

## 6. Conclusions

In this study, we presented a novel approach to passive frequency tuning in frequency-selective surfaces by utilizing Moiré interference patterns created through rotational misalignment of stacked periodic layers. By rotating one of two identical hexagonal FSS layers, we demonstrated, through full-wave simulations, that the effective resonance frequency can be continuously modulated over a wide range without any changes to the unit cell geometry or the addition of active components.

The simulation results confirmed that increasing the relative rotation angle from 0° to 30° shifts the resonance frequency from 4.4 GHz down to 1.2 GHz. This tunability is achieved solely through geometric manipulation, offering a low-complexity and power-free alternative to conventional active tuning methods. Furthermore, the observed narrowing of resonance peaks at higher rotation angles suggests improved selectivity, which can be advantageous in both filtering and sensing applications.

Although this work is limited to simulation-based analysis, it establishes a theoretical foundation for Moiré-based tunable FSS designs. Future research could extend this concept to include experimental validation, more complex geometries, tunable dielectric materials, and practical integration into optical and RF systems [34,55,56,57,58]. The proposed approach may find applications in reconfigurable filters, spectral sensors, and compact electromagnetic components where passive tunability is desirable.

## Data Availability

The original contributions presented in this study are included in the article. Further inquiries can be directed to the corresponding authors.
